# Comparison of Two Bayesian-MCMC Inversion Methods for Laboratory Infiltration and Field Irrigation Experiments

**DOI:** 10.3390/ijerph17031108

**Published:** 2020-02-10

**Authors:** Qinghua Guo, Fuchu Dai, Zhiqiang Zhao

**Affiliations:** Institute of Geotechnical Engineer, College of Architecture and Civil Engineering, Beijing University of Technology, Beijing 100124, China

**Keywords:** undisturbed loess, laboratory infiltration experiment, field irrigation experiment, Bayesian inversion, infiltration simulation

## Abstract

Bayesian parameter inversion approaches are dependent on the original forward models linking subsurface physical properties to measured data, which usually require a large number of iterations. Fast alternative systems to forward models are commonly employed to make the stochastic inversion problem computationally tractable. This paper compared the effect of the original forward model constructed by the HYDRUS-1D software and two different approximations: the Artificial Neural Network (ANN) alternative system and the Gaussian Process (GP) surrogate system. The model error of the ANN was quantified using a principal component analysis, while the model error of the GP was measured using its own variance. There were two groups of measured pressure head data of undisturbed loess for parameter inversion: one group was obtained from a laboratory soil column infiltration experiment and the other was derived from a field irrigation experiment. Strong correlations between the pressure head values simulated by random posterior samples indicated that the approximate forward models are reliable enough to be included in the Bayesian inversion framework. The approximate forward models significantly improved the inversion efficiency by comparing the observed and the optimized results with a similar accuracy. In conclusion, surrogates can be considered when the forward models are strongly nonlinear and the computational costs are prohibitive.

## 1. Introduction

Appropriate determination of model parameters is important to obtaining accurate simulations of a hydrological system. The stochastic inversion method, which updates the distribution of model parameters using the observed state variables, has the advantage of quantifying parameter uncertainty [1]. Bayesian inversion of hydrological and geophysical parameters combined with Markov Chain Monte Carlo (MCMC) sampling methods has become increasingly popular in recent decades [2,3]. However, in the Bayesian-MCMC approach, a large number of forward model runs is commonly required to acquire meaningful posterior statistics, which is computationally prohibitive for many real-world applications [4]. A variety of techniques can be applied to reduce the computational load of Bayesian-MCMC inversion and improve the analysis efficiency [5,6]. Among them, the most intuitive and commonly employed method is the application of surrogates of the original forward solvers [7,8]. The data-driven alternative systems constructed by the input and output samples of the original forward models are widely applied for numerical simulation and uncertainty quantification.

In the subsurface hydrologic field, various approximations of the original forward models are introduced to improve the computational efficiency of the Bayesian-MCMC approach. However, the discrepancies between the approximated and original forward models will affect the simulation quality if they are not taken into account [9]. Neglecting these discrepancies can lead to strongly biased and overconfident posterior distributions and subsequently affect future predictions [10]. To improve the performance of approximate forward solvers during Bayesian calibration, a number of approaches has been developed to account for model errors in recent years [11,12,13]. Model error representations can be classified as either external bias descriptions or internal noise descriptions [14]. Generally, the discrepancy between approximate and detailed forward solvers in Bayesian stochastic inversions can also be classified into two categories. In the first category, researchers have focused on the overall statistics of the model error with the goal of developing more appropriate parametric likelihood functions that better reflect the true nature of the residual [15]. By far the most representative approach is to assume that the model errors follow a Gaussian distribution, indicating that the means and covariances estimated from the realizations can be incorporated into the Bayesian likelihood functions [16,17,18]. It has been shown that the inclusion of model-error statistical characteristics into the Bayesian likelihood function leads to broadened posterior distributions and lower parameter bias. In the second category of developed approaches, to account for the model error component induced by the approximate system, researchers have focused on building error model systems to describe the discrepancy between the full and approximate forward solutions over the model parameter space [19]. However, in this category, the results are used to correct the output of the approximate solver rather than to develop a more approximate Bayesian likelihood function. For example, the model-error component can be obtained by applying a local orthonormal basis generated by the K-nearest neighbours in an error dictionary that is continuously enriched during the calibration process [20]. The principal component analysis (PCA) is also used to quantify the model error [21,22].

This paper aims to examine the performances of the Artificial Neural Network (ANN) and Gaussian Process (GP) surrogate systems by considering the model errors arising from the use of approximate forward solvers with different ways to simulate the pressure heads of the undisturbed loess obtained from infiltration experiments. The rest of the paper is organized as follows. Section 2 provides a detailed description of the proposed methods. Following that, two case studies with detailed experiment descriptions and special parameters for the proposed algorithms are presented in Section 3. Section 4 displays the results and Section 5 displays the discussion. Section 6 presents an overall assessment and the conclusions of the work.

## 2. Materials and Methods

### 2.1. Basic Theory

The Bayesian approach used in this paper is a recently popular stochastic method which is based on Bayesian principle:

(1) p(m|d)∝p(m) p(d|m),

where p(m) is the prior distribution of the model parameters, which is the understanding of the model parameters before obtaining the observed value d. p(m|d) is a posterior distribution of the model parameters, which is updated by the observed value d. p(d|m)≡L(m|d) is the likelihood function, which is a measure of the proximity between the model output F(m) and the observed value d. The closer F(m) and d are, the greater the value of p(d|m).

The governing equation describing the process of soil water movement is the one-dimensional Richards equation, whose form is as follows:

(2) $$\frac{\partial \theta \left(h\right)}{\partial t}=\;\frac{\partial }{\;\partial z}\;\left[K\left(h\right)\;\frac{\partial h}{\partial z}\;-\;K\left(h\right)\right],\;$$

where θ is the volumetric water content [$L^{3}{\;L}^{-3}$]; h is the pressure head [L]; K(h) is the unsaturated hydraulic conductivity $\;[{L\;T}^{-1}$]; z is the vertical depth [L]; t is the time [T]. The relationship between water content and pressure head, as well as the relationship between unsaturated hydraulic conductivity and pressure head, is described by the Van Genuchten-Mualem (VGM) model.

(3) $$\theta \left(h\right)=\{\begin{array}{c} \theta_{r}+\frac{\theta_{s}-\theta_{r}}{{\left(1+{\left|\alpha h\right|}^{n}\right)}^{m}},\;\;h<0\\ \theta_{s},\;\;\;\;\;\;\;\;\;h\geq 0 \end{array},$$

(4) $$K\left(h\right)=\;\{\begin{array}{c} K_{s}S_{e}{}^{1/2}{\left[1-{\left(1-S_{e}{}^{1/m}\right)}^{m}\right]}^{2},\;\;\;\;\;\;\;\;h<0\\ K_{s},\;\;\;h\geq 0\;\;\;\;\;\;\;\;\; \end{array},$$

(5) $$S_{e}=\frac{\theta -\theta_{r}}{\theta_{s}-\theta_{r}},$$

m = 1 − 1/n,(6)

where $S_{e}$ is the effective saturation; α is the parameter related to the average particle size; n is the parameter related to the particle size uniformity; $\theta_{s}$ is the saturated water content; $\theta_{r}$ is the residual water content; $K_{s}$ is the saturated water conductivity. From the above analysis, it can be seen that the parameters needing to be inverted are $\theta_{s}$, $\theta_{r}$, *α*, *n*, $K_{s}$.

### 2.2. ANN Appropriate System

#### 2.2.1. ANN Optimized by the Particle Swarm Optimization (PSO) Algorithm

**Figure 1:**
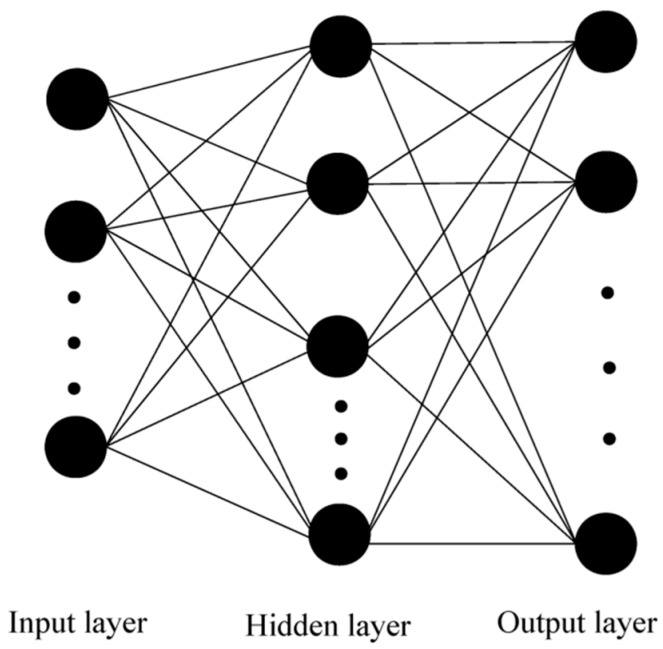
Typical structure of an Artificial Neural Network composed of three layers.

The ANN is a machine learning algorithm that is well known and widely applied in many fields [23]. It depends only on the data and learns from them without any predefined analytical and statistical dependence assumptions. The Back Propagation (BP) neural network is commonly thought of as providing universal approximations for the function mapping whose training process includes the forward propagation of signals and the reverse propagation of errors. As displayed in Figure 1, the structure of an ANN generally consists of an input layer, a hidden layer and an output layer. The relationship between the different layers of the ANN can be expressed as follows [24]:

(7) $$y_{j}=\;f\;\left(\sum_{i=1}^{N}\omega_{\mathrm{ji}}x_{i}+b_{j}\right),$$

where $y_{j}$ is the value of the $j^{\mathrm{th}}$ node in the current layer, f (.) is the activation function in the current layer, $x_{i}$ is the value of the $i^{\mathrm{th}}$ node in the previous layer, $\omega_{\mathrm{ji}}$ is the weight connecting $x_{i}$ and $y_{j}$, N is the number of nodes in the previous layer, and $b_{j}$ is the threshold of the $j^{\mathrm{th}}\;$ node in the current layer. The input layer contains the input parameters of the model. The output layer contains the desired outputs of the system and the hidden layer usually contains a series of nodes associated with transfer functions. The Levenberg–Marquardt algorithm based on the gradient descent and quasi-Newton methods is applied to modify the weights and thresholds of neural networks. In the groundwater field, the ANN is widely adopted to construct alternative systems, such as those for multiphase flow simulation and groundwater level prediction [25]. In addition, a PSO algorithm with von Neumann structure is utilized to optimize the original neural network. PSO is a type of swarm intelligence algorithm used for parameter optimization problems [26]. The von Neumann structure is a spatial structure formed by connecting the vertices of a square mesh, which is the best topological structure used in particle swarm algorithms with respect to optimization speed and ability. In the structure, the particles exchange information with the four directly connected particles, that is, the neighbourhood’s optimal particle uses the best of the four directly connected particles.

#### 2.2.2. Procedures for the ANN-Based Bayesian-MCMC Algorithm

The ANN and PSO can be combined in the parameter inversion calculation process to simplify the analytical process. PCA can identify and remove the model errors introduced by the application of the ANN approximate forward model before the calculation of the likelihood function in the DREAMzs algorithm [27,28]. The detailed procedure of the algorithm is given as follows:

1. Generate k random sets of model parameters $M\;=\;\{m_{1},\ldots ,{\;m}_{k}$} from the parameter prior distributions. Compute the corresponding sets of simulated output results $F\;=\;\{F\left(m_{1}\right),\;\ldots ,\;F(m_{k})\}$ using the original HYDRUS-1D forward model.
2. Construct a three-layer BP neural network in which the number of the unknown hydraulic parameters is the number of input layer nodes and the number of measured pressure heads during the experiments is the number of output layer nodes. A number of initial weights and threshold combinations are randomly generated using the data pairs {M, F}.
3. Optimize the weights and thresholds of the neural network using the PSO algorithm, where each combination obtained in the above step is considered to be a particle. After that, the optimal neural network representing the forward analysis process is achieved.
4. Compute the corresponding sets of stochastic model-error realizations $\;E\;=\;\{E_{1},\ldots ,E_{k}$}, where $E_{i}=\;F\left(m_{i}\right)\;-\hat{F}\left(m_{i}\right)$, $\hat{F}(m_{i}$) is the corresponding output results of the ANN approximate system formed in the above step, F($m_{i}$) is those of the original HYDRUS-1D forward model, and i = 1,…,k.
5. Perform PCA on the model-error realizations $\;E\;=\;\{E_{1},\ldots ,E_{k}$} to obtain a sparse orthonormal basis $\;B\;=\;\{b_{1},\ldots ,b_{b}$} for the model error. For each set of model parameters m′ tested within the MCMC, the model error component of the discrepancy between the measured values and the ANN simulated values is obtained by projecting the discrepancy to the orthonormal basis B.
6. The model error component received above is subtracted from the corresponding residual of the Bayesian likelihood function. Run the Bayesian-MCMC algorithm using the ANN instead of the original HYDRUS-1D forward model to generate samples of the posterior distribution.

### 2.3. Basic Theory of the Gaussian Process

The GP is a supervised learning algorithm which can construct a stochastic system based on N groups of input and output samples from the original models (called base points here):

(8) G(m)~N(μ(.),k(.,.)),

where μ(.) is the mean function which can be used as the output of the alternative system; and k(.,.) is the covariance function, which can quantify the output uncertainty of the alternative system. The mean function can be a constant, linear or polynomial function, while the covariance function can be a linear, polynomial or square exponential function. The zero-mean function and the squared exponential covariance function are applied in this study. After determining the form of the mean and covariance functions, the hyper-parameters of these functions are optimized utilizing the maximum likelihood ratio method. Given the optimal hyper-parameters, the conditional GP mean $u_{|Y}\left(u\right)$ and variance $\sigma_{|Y}^{2}\left(u\right)\;$ for an arbitrary parameter sample u can be obtained. The GP surrogate can easily be coupled with the Bayesian-MCMC parameter inversion algorithm and you can refer to [29] for the construction of the GP-based inversion framework.

## 3. Case Studies

Extensive agricultural irrigation in the South Jingyang Platform of Northwest China has seriously damaged the local hydrogeological environment and induced a large number of landslides [30,31]. To properly understand the hydrological processes and the hydraulic properties of the corresponding undisturbed loess, a field undisturbed loess irrigation test and a laboratory soil column infiltration test were carried out. Based on the measured pressure head values, as well as the prior distribution of the soil hydraulic parameters, the adaptive Bayesian-MCMC simulation was implemented to infer the model parameters. The posterior distribution is regarded as stationary if the $\hat{R}$-statistic proposed by Gelman and Rubin is less than 1.2 [32]. In both cases, the MCMC sampler was performed with three simultaneous chains for a total number of 50,000 runs. Depending on the scenario, the MCMC required approximately 25,000–30,000 model runs to converge. For each case, the different results obtained from the Bayesian-MCMC frameworks based on the original HYDRUS-1D forward model, the ANN approximate forward model and the GP approximate forward model were compared.

### 3.1. Case 1: Laboratory Infiltration Experiment

#### 3.1.1. Obtainment of the Measurement Data

**Figure 2:**
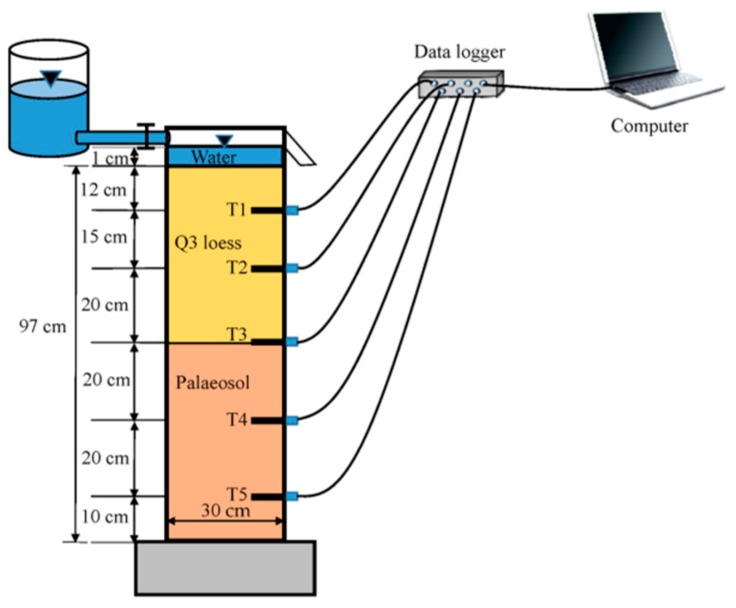
Instruments for the laboratory infiltration experiment.

The undisturbed loess column was collected by carefully introducing a rigid polyvinyl chloride (PVC) cylinder into the soil. The PVC cylinder was 100 cm in height and 30 cm in inner diameter with a wall thickness of 1 cm. Three centimeters of soil was removed from the top of the column to eliminate the influence of the disturbed soil, leaving 47 cm of Q3 loess and 50 cm of S1 palaeosol. The soil column was equipped with five tensiometers (T1 to T5) 12, 27, 47, 67, and 87 cm from the surface of the soil column, respectively (Figure 2). The water supply container was connected and an overflow hole was provided 2 cm from the top of the PVC cylinder to maintain a 1 cm constant water head. The data acquisition interval was 1 min. The experiment ended after 575 min when the response of the fifth tensiometer was terminated.

#### 3.1.2. Construction of the Original HYDRUS model and Surrogate Systems

In the original HYDRUS model, the soil column is vertically divided into 97 units with an interval of 1 cm. As a result, there are 98 nodes in total. The whole simulated soil layer is divided into two sub areas for the reason that the top 47 cm of the soil is Q3 loess, and the bottom 50 cm is S1 palaeosol. Five sensor installation depths (12 cm, 27 cm, 47 cm, 67 cm and 87 cm from the surface of the soil column) are set as observation points. The length unit is selected as centimeter and the time unit is set as minute. The soil pressure head at the time t = 0 min was used as the initial condition to simulate the soil water movement process of soil column. According to the initial values of the five sensors, the initial pressure head of 98 nodes is obtained by interpolation. The upper boundary is 2 cm constant head boundary, and the lower boundary is set as free drainage boundary. In the construction of the ANN approximate system, k = 1200 design points, $M\;=\;\{m_{1},\;\ldots ,{\;m}_{k}$}, were empirically drawn from the prior parameter distributions to achieve the initial training data, $F=\{F\left(m_{1}\right),\ldots ,\;F(m_{k})\}$. After that, the ANN surrogate was trained conditioned on [M F] and applied to accelerate the MCMC simulation using the DREAMzs algorithm [3]. To accelerate the construction process, the k design points whose model outputs were closer to the measurements were used to construct the ANN surrogate. The construction of the GP surrogate is similar to that of the ANN mentioned above except that the number of basic points is k = 200. There are five unknown parameters for each layer; as a result, there are ten input values from both the Q3 loess and palaeosol soil layers. As mentioned above, the infiltration experiment lasted 575 min recording the data once a minute. To simplify the calculation, one out of every 40 data was chosen for each tensiometer. That is to say that the number of the observed pressure head values used in the simulation is 75.

### 3.2. Case 2: The Field Irrigation Experiment

#### 3.2.1. Obtainment of the Measurement Data

**Figure 3:**
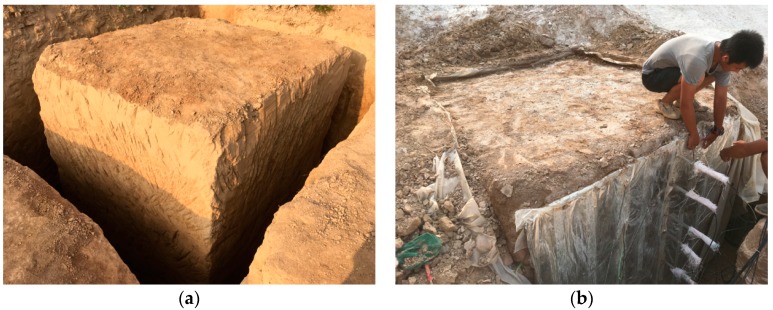
Field irrigation experiment: (**a**) field undisturbed loess column; (**b**) instrument of tensiometers.

**Figure 4:**
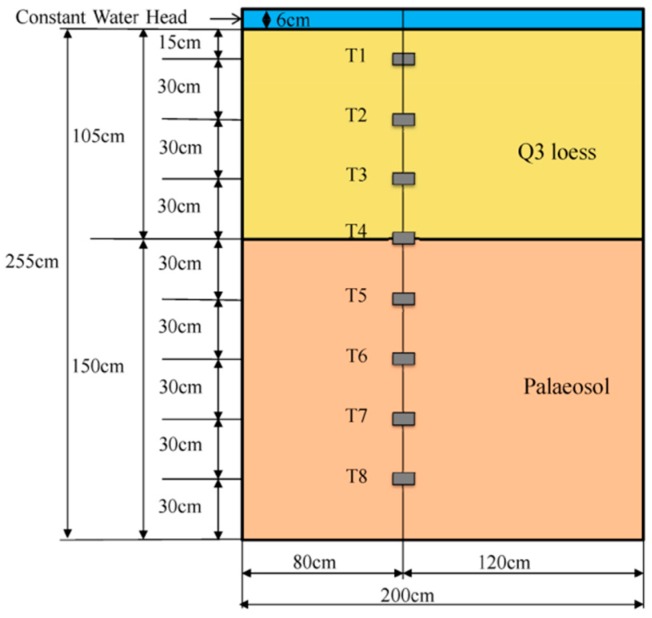
Instruments for the field irrigation experiment.

A soil column with a length and width of 200 cm and a height of 255 cm was manually excavated, then was surrounded and sealed by bentonite and plastic film (Figure 3). The soil column was composed of two different soil layers, the upper 105 cm Q3 loess and the lower 150 cm S1 palaeosol. Eight tensiometers (T1 to T8) were embedded along the depth of the soil column and the corresponding depths were 15 cm, 45 cm, 75 cm, 105 cm, 135 cm, 165 cm, 195 cm, and 225 cm, respectively (Figure 4), from the top surface of the soil column. A water retaining plate was arranged above the soil column to provide a 6 cm constant head boundary condition during the irrigation process. The irrigation test stopped when the tensiometer at the 225 cm depth responded, which happened after 58.65 h. The data acquisition interval was 3 min and 1174 reads for each sensor.

#### 3.2.2. Construction of the Original HYDRUS Model and Surrogate Systems

In the original HYDRUS model, the soil column is vertically divided into 255 units with an interval of 1 cm. As a result, there are 256 nodes in total. The whole simulated soil layer is divided into two sub areas for the reason that the top 105 cm of the soil is Q3 loess, and the bottom 150 cm is S1 palaeosol. Eight sensor installation depths (15 cm, 45 cm, 75 cm, 105 cm, 135 cm, 165 cm, 195 cm, and 225 cm from the surface of the soil column) are set as observation points. The length unit is selected as centimeter and the time unit is set as minute. The soil pressure head at the time t = 0 min was used as the initial condition to simulate the soil water movement process of the soil column. According to the initial values of the eight sensors, the initial pressure head of 256 nodes is obtained by interpolation. The upper boundary is 6 cm constant head boundary and the lower boundary is set as free drainage boundary. To simplify the calculation, one out of every 60 data of the 4th and 5th tensiometers were chosen to verify the performances of the proposed inversion methods. Thus, the number of observed pressure head values used in the simulation is 118. The construction of approximate systems is similar to the description in Section 3.1.2 with the exception of the number of calibration measurement values. Moreover, in the construction of the ANN approximate system, k = 1600 design points were empirically drawn to satisfy the condition that the number of the training samples should be larger than that of the weight values and thresholds.

## 4. Results

### 4.1. Case 1 Results

Table 1 shows the ranges and the maximum a posteriori (MAP) inversed values derived from three different methods of the ten hydraulic parameters for the laboratory infiltration experiment. The different methods do not show a large difference in optimal parameter values. The results reveal that the surrogates can yield MAP parameter estimates without marked bias compared to those obtained with the original forward model at reasonable computational costs.

**Figure 5:**
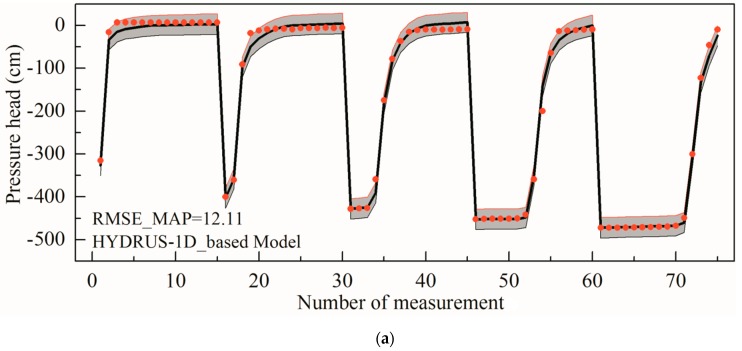

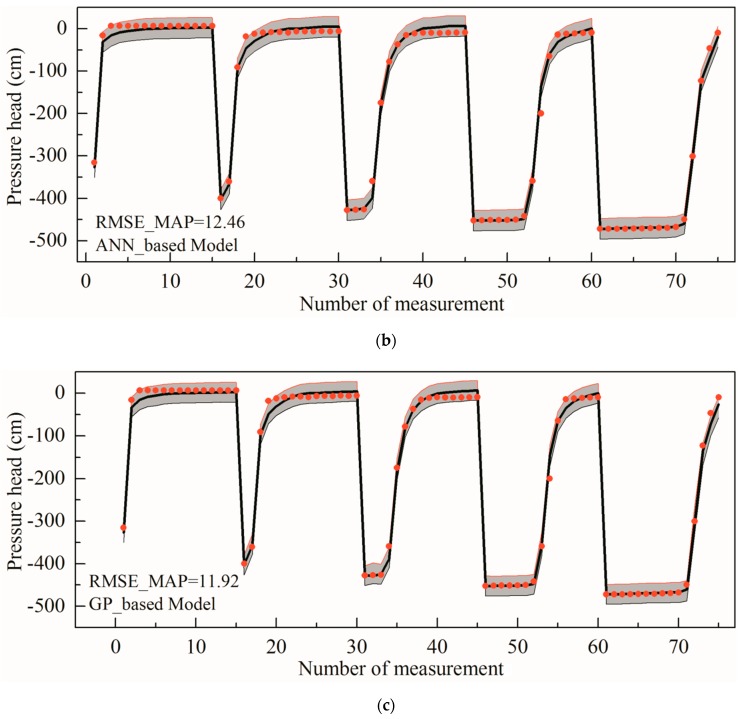
Results of the laboratory infiltration experiment. The red dots are the 75 pressure head measurement values and the grey areas are the 95% confidence intervals of the MCMC simulation results. The solid lines denote the simulated results using the posterior MAP parameters. (**a**) Results based on Hydrus-1D model (**b**) Results based on ANN model (**c**) Results based on GP model

The average time to execute the original Hydrus-1D model once was 2 s. As a consequence, the MCMC simulation with the original model demanded approximately 27.78 h for 50,000 repetitions. The time needed by the ANN and GP-based Bayesian inversions is approximately 8 h and 13 h, respectively, which includes the surrogate construction time and the MCMC algorithm running time. Thus, the approximate system-based MCMC simulations are much more efficient than the original model-based MCMC simulation. The results of the Bayesian-MCMC inversions using three different forward models were compared. The first one is the result of the original forward model based Bayesian-MCMC method (Figure 5a), which is followed by the simulation result of an ANN surrogate combined with the PCA correction of the model error described in Section 2.1 (Figure 5b). Finally, the one derived from the case using the GP approximate system was illustrated (Figure 5c). The Root Mean Square Error_Maximum a posteriori (RMSE_MAP) values of the three different methods are similar at 12.11, 12.46 and 11.92, respectively, which indicates that the match between the MAP simulated outputs and the observed data of the experiment is good. A good agreement can also be observed between the black line and the red dots illustrated in Figure 5. It can also be confirmed that the approximate systems are reliable enough to reproduce the infiltration experiment of the investigated undisturbed soil, which is consistent with Table 1. Figure 5 shows that most of the experimental data can be covered by the 95% confidence intervals, indicating a good uncertainty quantification obtained by the Bayesian estimation.

### 4.2. Case 2 Results

Table 2 shows the ranges and the MAP inversed values derived from three different methods of the ten hydraulic parameters for the field irrigation experiment. The different methods do not show a large difference in optimal parameter values. The results reveal that the surrogates can yield MAP parameter estimates without marked bias compared to those obtained with the original forward model at reasonable computational costs.

**Figure 6:**
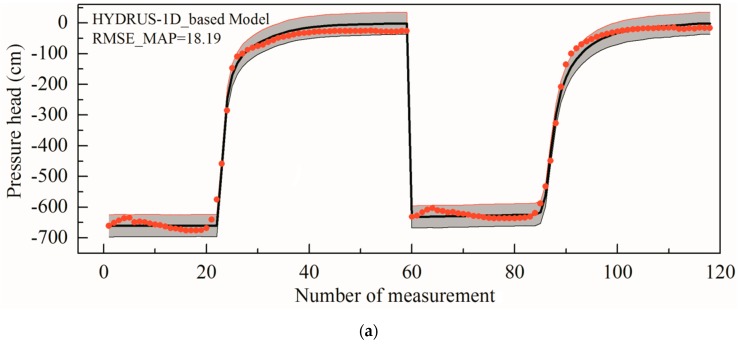

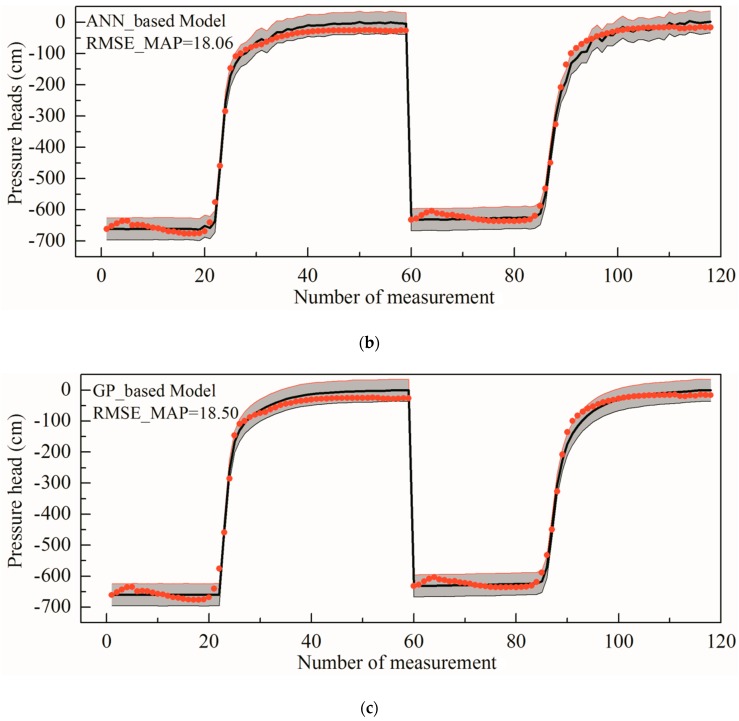
Results for the field irrigation experiment. The red dots are the 75 pressure head measurement values and the grey areas are the 95% confidence intervals of the MCMC simulation results. The solid lines denote the simulated results using the posterior MAP parameters. (**a**) Results based on Hydrus-1D model (**b**) Results based on ANN model (**c**) Results based on GP model

It takes approximately 8 s to perform the original Hydrus-1D model once. Consequently, the original model based MCMC simulation took approximately 4.6 days to draw the 50,000 chain states. The times needed by the ANN and GP-based Bayesian inversions are approximately 23 h and 19 h, respectively, which included the surrogate construction time and the MCMC algorithm sampling time. Thus, the approximate system-based MCMC simulations are much more efficient than the original model-based MCMC simulation. The RMSE_MAP values of the three different methods are 18.5, 18.06 and 18.19, respectively, implying that the matches between the predictions using the MAPs and the experimental data are good. This is consistent with Figure 6, in which a good agreement can also be observed between the black lines and the red dots. Figure 6a exhibited the results of the original forward model based Bayesian-MCMC method, which is followed by the simulation results of an ANN surrogate combined with the PCA correction for the model error described in Section 2.1 (Figure 6b). Finally, the results derived from the case applying the GP approximate system was displayed (Figure 6c). Most of the experimental data can be covered by the 95% confidence intervals, indicating a good quantification of the uncertainty by the Bayesian estimation. It can be seen that the RMSE_MAPs of the field irrigation experiment are larger than those of the laboratory soil column infiltration experiment, for the reason that the field irrigation conditions are more complex and the observation accuracy is lower than those of the laboratory infiltration experiment.

## 5. Discussion

From the above two cases, it can be seen that the application of alternative systems does apparently improve the calculation efficiency while not differing much in performance, but they also have several shortcomings. It is difficult to completely eliminate the model error and obtain a completely unbiased estimation (Table 1 and Table 2), especially when the calibration data are acquired from the undisturbed structural loess experiment. The outputs of the training samples should be close to the experimental observation values to efficiently obtain an ideal alternative system; therefore, the selection of the training samples also takes a certain amount of time. Moreover, the substitute systems can only predict the fixed sample elements used in the training of the mapping relationships while the original model can verify any desired input and output variables without any limitations. In addition, when using the substitute systems for parameter inversion, most of the time is spent on training the substitute system since the run of the substitute system once is very fast. The number of training samples and the number of elements in each sample will strongly affect the training times of the substitute systems, especially the GP for which the calculation costs will cubically increase as the number of samples increases [33]. In conclusion, the original forward model can be used as much as possible when it is relatively simple. Otherwise, the alternative systems can be applied to improve the calculation efficiency while ensuring a certain degree of accuracy. Therefore, the approximations can be used by trading-off accuracy for speed [34].

Both the ANN and GP surrogates are data-driven approximate systems that construct the mapping relationships based on the input-output samples generated from the original HYDRUS-1D forward model. The first difference between them is the ways in which they handle the discrepancy between the surrogate and the original model. The discrepancy introduced by the ANN approximation should be identified and removed from the residual using the orthonormal basis generated by PCA before the calculation of the Bayesian likelihood function in each iteration [21]. However, the GP could automatically derive the statistical covariance of the randomly drew parameter sample and directly include the corresponding statistical covariance in the likelihood function of each iteration [29]. This difference leads to a relatively more complex and more time consuming MCMC sampling process of the ANN-based model compared with that of the GP-based model. The second difference between them is that the number of training samples theoretically needed is different. The number of training samples should be larger than the total number of the weight and the threshold values in the ANN approximate system, while the number of training samples should be larger than the number of the hyper-parameters in the mean and covariance functions of the GP surrogate system. In this study, the number of input-output samples used in the ANN training process was larger than that used in the GP. However, the iteration time of the ANN training is much less than that of GP, which led to the results that the total time used to training the ANN system is not quite different from that used to training the GP system.

## 6. Conclusions

In this paper, the application of approximate forward models, namely, the ANN and GP, were examined to improve the efficiency of the Bayesian-MCMC approach combined with the observed pressure head values. The main conclusions are as follows:

1. Approximate system-based MCMC methods can considerably accelerate the inverse modelling in layered loess since the computational cost of the surrogate-based MCMC simulation is rather lower. Furthermore, the optimal parameter values derived from different methods do not show a large difference, which signifies that the surrogates can yield MAP parameter estimates without marked bias compared to those obtained with the original HYDRUS model at reasonable computational costs.
2. The simulated pressure head values in the layered undisturbed loess profile are consistent with the experimental results. Specifically, the simulations using the MAP parameter values are extremely close to the experimental data, which also fall in the 95% posterior confidence intervals. Uncertainty analyses of the pressure heads supported the reliability of the ANN/GP-based MCMC. The fitting effect of the field test is not as good as that of the indoor test since there are more complex conditions in the field.
3. The similar RMSE_MAPs of both the ANN and GP approximate systems indicates that they offer almost equally good performances. These results are, of course, related to the models and special experimental conditions considered. The suggested methods should be verified and improved for different types of soils and more complex conditions.
4. The main shortcoming of the suggested surrogate methods is that the construction time of the approximate system is strongly related to the number of training samples. The computational costs of the GP construction cubically increase as the amount of training data increases. For a high-dimensional and complex system, training ANN/GP surrogates would be extremely time-consuming. Moreover, the substitute systems can only predict the fixed elements used in the training samples of mapping relationships.

## Figures and Tables

**Table 1:** Parameter ranges and MAP inversed by three different methods.

Type of Parameters		$\theta_{r}\;$	$\theta_{s}\;$	α	*n*	*K_s_* (m/min)
Q3	Parameter Ranges	[0.15, 0.2]	[0.45,0.55]	[0.003, 0.01]	[1.5, 2.5]	[0.005, 0.02]
	HYDRUS-Based-MAP	0.1702	0.4791	0.0063	1.5003	0.0156
	ANN-Based-MAP	0.2	0.45	0.0044	1.5031	0.02
	GP-Based-MAP	0.1719	0.4795	0.0099	1.5199	0.0154
S1	Parameter Ranges	[0.15, 0.2]	[0.45, 0.55]	[0.003, 0.01]	[1.5, 2.5]	[0.005, 0.015]
	HYDRUS-Based-MAP	0.2	0.45	0.0073	1.5	0.01
	ANN-Based-MAP	0.1997	0.45	0.0094	1.6227	0.01
	GP-Based-MAP	0.1993	0.4505	0.0095	2.1504	0.0067

**Table 2:** Parameter ranges and MAP inversed by three different methods.

Type of Parameters		$\theta_{r}\;$	$\theta_{s}\;$	α	*n*	*K_s_* (m/min)
Q3	Parameter Ranges	[0.15, 0.2]	[0.45,0.55]	[0.003, 0.01]	[1.5, 2.5]	[0.005, 0.02]
	HYDRUS-Based-MAP	0.1906	0.4638	0.0097	2.4997	0.0087
	ANN-Based-MAP	0.15	0.45	0.0065	1.585	0.0088
	GP-Based-MAP	0.1999	0.4693	0.003	2.5	0.008
S1	Parameter Ranges	[0.15, 0.2]	[0.45,0.55]	[0.003, 0.01]	[1.5, 2.5]	[0.005, 0.015]
	HYDRUS-Based-MAP	0.2	0.45	0.0047	1.6335	0.01
	ANN-Based-MAP	0.2	0.45	0.0034	1.5199	0.008
	GP-Based-MAP	0.2	0.4586	0.003	1.6205	0.005
